# Quorum Sensing in *Bacillus thuringiensis* Is Required for Completion of a Full Infectious Cycle in the Insect

**DOI:** 10.3390/toxins6082239

**Published:** 2014-07-31

**Authors:** Leyla Slamti, Stéphane Perchat, Eugénie Huillet, Didier Lereclus

**Affiliations:** INRA, Unité MICALIS UMR-1319, La Minière, 78280 Guyancourt, France; AgroParisTech, UMR MICALIS, F-78352 Jouy-en-Josas, France; E-Mails: stephane.perchat@jouy.inra.fr (S.P.); eugenie.huillet@jouy.inra.fr (E.H.); didier.lereclus@jouy.inra.fr (D.L.)

**Keywords:** RNPP, cell-cell communication, *Bacillus thuringiensis*, adaptation, virulence, necrotrophism, sporulation, gene expression

## Abstract

Bacterial cell-cell communication or quorum sensing (QS) is a biological process commonly described as allowing bacteria belonging to a same pherotype to coordinate gene expression to cell density. In Gram-positive bacteria, cell-cell communication mainly relies on cytoplasmic sensors regulated by secreted and re-imported signaling peptides. The *Bacillus* quorum sensors Rap, NprR, and PlcR were previously identified as the first members of a new protein family called RNPP. Except for the Rap proteins, these RNPP regulators are transcription factors that directly regulate gene expression. QS regulates important biological functions in bacteria of the *Bacillus cereus* group. PlcR was first characterized as the main regulator of virulence in *B. thuringiensis* and *B. cereus*. More recently, the PlcR-like regulator PlcRa was characterized for its role in cysteine metabolism and in resistance to oxidative stress. The NprR regulator controls the necrotrophic properties allowing the bacteria to survive in the infected host. The Rap proteins negatively affect sporulation via their interaction with a phosphorelay protein involved in the activation of Spo0A, the master regulator of this differentiation pathway. In this review we aim at providing a complete picture of the QS systems that are sequentially activated during the lifecycle of *B. cereus* and *B. thuringiensis* in an insect model of infection.

## 1. Introduction

*Bacillus thuringiensis* produces insect-specific delta-endotoxins (or Cry proteins) that form a crystal in the bacterium during growth under nutrient starvation [1]. The entomopathogenic properties of this bacterium are widely used in the world for pest control [2,3]. These beneficial properties should not hide the fact that, apart from the production of crystal toxins, *B. thuringiensis* is phenotypically and genetically indistinguishable from *B. cereus* [4], a human pathogen responsible for foodborne toxi-infections [5]. The emetic syndrome in these infections is caused by the cereulide peptide produced by some strains [6,7]. *B. cereus* has also been described as the cause of nosocomial infections in immuno-compromised patients and was involved in endocarditis, endophtalmites or meningitis that have proved fatal [8,9,10,11]. This bacterium produces an arsenal of factors that are likely to be responsible for its opportunistic properties. These invasins allow the destruction of tissues and the multiplication of the bacteria in their host and are shared by *B. thuringiensis*. Among them, the phosphatidylinositol phospholipase C PI-PLC, the non-hemolytic enterotoxin Nhe and the hemolysins Hbl, CerAB and CytK are found. CytK, Hbl and Nhe were also linked to toxi-infections with a diarrheic syndrome [5]. The genes encoding these proteins are under the control of the transcription regulator PlcR and thus belong to the PlcR regulon [12]. A *B. thuringiensis* or a *B. cereus* mutant devoid of PlcR will not succeed in infecting its host in an insect or a mouse model of infection [13]. These mutants are also significantly attenuated in an endophtalmitis model of infection [14]. 

Besides its virulent behavior, a recent report highlighted a new lifestyle for *B. thuringiensis* in insects [15]. *B. cereus* and *B. thuringiensis* are capable of multiplying in insects, which is a privileged ecological niche for both bacteria [16]. It was shown that the *B. thuringiensis* genome contains a set of genes specifically induced after the insect death [15]. Expression of these genes, encoding degradative enzymes, such as metalloproteases, lipases, and chitinases, but also the enzymatic machinery synthesizing the lipopeptide kurstakin, depends on the transcription activator NprR. This regulator is responsible for the necrotrophic behavior of *B. thuringiensis* in its host as an *nprR* deletion mutant does not survive in the insect cadaver. Necrotrophism may allow *B. thuringiensis* to multiply and delay sporulation, which is a costly, irreversible, but necessary process to ensure proper dissemination of the bacteria in the environment.

The spore is an ultimate form of resistance and is generally regarded as a potent infectious agent. It is the main factor responsible for dispersal and survival of *B. thuringiensis* and *B. thuringiensis* in various environments including the digestive tract, food or industrial equipment. Sporulation is under the control of the master regulator Spo0A-P [17]. In the model organism *Bacillus subtilis*, environmental cues lead to the transduction of a signal inducing the phosphorylation of Spo0A via several histidine kinases and the phosphorelay proteins Spo0F and Spo0B [18]. Spo0A-P can then either activate or repress the transcription of genes by binding to the 0A box [19]. Deletion of *spo0A* affects several pathways but most notably renders *B. thuringiensis* and *B. cereus* incapable of initiating sporulation [20].

**Figure 1:**
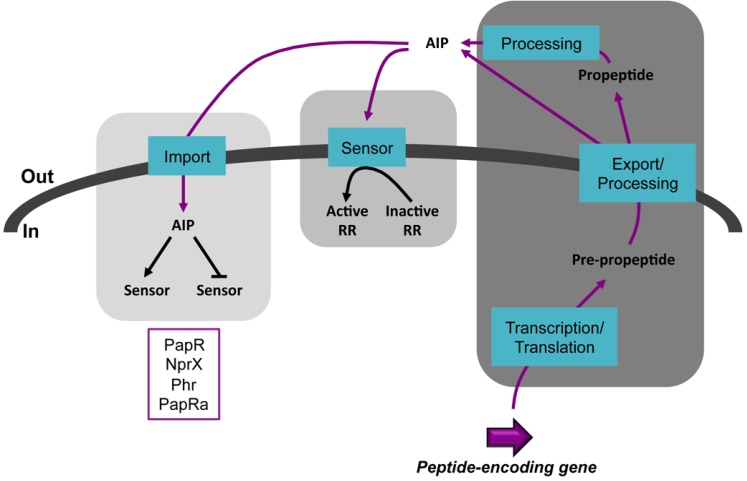
Schematic representation of the two types of QS systems involving AIP in Gram-positive bacteria. The steps common to the two systems are on the right. The middle panel represents the extracellular-acting AIP-dependent mechanism. The left panel represents the intracellular-acting AIP-dependent mechanism. This study will focus on QS systems relying on the peptides indicated in the purple box. Black arrows represent a positive effect. Black blunt lines represent a negative effect. Purple lines indicate the circuit followed by the AIP. RR, response regulator.

The expression of virulence, necrotrophism and sporulation genes depends on quorum sensing (QS) systems in *B. cereus* and *B. thuringiensis*. QS relies on small secreted molecules known as autoinducers (AI) or pheromones. The three most studied kinds of QS systems involve acyl-homoserine lactones (AI-1) used by Gram-negative bacteria; small oligopeptides (AIP) used by Gram-positive bacteria and cyclic furanone-based compounds (AI-2) used by both Gram-negative and Gram-positive bacteria [21,22]. The peptide-based Gram-positive systems can be further classified into two categories depending if the target of the peptide is outside or inside the cell (Figure 1). Extracellular-acting AIP are recognized by a membrane-embeded histidine kinase that will phosphorylate a cognate response regulator upon peptide binding. This will activate the response regulator, enabling it to regulate the expression of target genes. A well-studied example of this type of QS is the Agr system of *Staphylococcus aureus* that controls the expression of virulence genes [23,24]. Intracellular acting AIP are internalized in the cell via an oligopeptide permease system. They will then bind to a sensor thereby affecting its activity as a transcriptional regulator or rendering it inactive by disrupting its interaction with another protein. The former case is exemplified by the PrgX-cCF10 system that controls conjugation in *Enterococcus faecalis* [25,26,27] and the latter by the Rap-Phr pairs involved in sporulation and competence in *B. subtilis* [28,29,30]. Common features of the extracellular- and intracellular-acting peptides are that they are genetically encoded and ribosomally translated, actively secreted and processed with some differences for these two latter steps.

The research field of intracellular-acting communication systems has been recently marked with several important milestones, notably with the structural characterization of some of the above mentioned regulators with their interacting partners [31,32,33,34,35,36,37]. The structural features of the Rap, NprR, PlcR, and PrgX quorum sensors allowed their classification as members of a new protein family called RNPP [32]. These quorum sensors are characterized by the presence of six to nine tetratricopeptide repeats (TPR) forming the peptide-binding domain. Except for the Rap proteins, they also contain an N-terminal helix-turn-helix (HTH) DNA-binding domain.

The role, regulation and interconnection of these RNPP QS systems in *B. thuringiensis* during infection of insect larvae will be the focus of this review.

## 2. Kill: PlcR and Virulence

PlcR was first identified in *B. thuringiensis* as the transcriptional activator of the *plcA* gene, which encodes the phosphatidylinositol-specific phospholipase C (PI-PLC) [38]. PlcR activates its own transcription and that of *plcA* at the end of the exponential growth phase. A genetic screen demonstrated that PlcR is a pleiotropic regulator that controls the transcription of several genes encoding exported proteins putatively involved in virulence [39]. As mentioned in the introduction, these factors include various degradative enzymes and cytotoxins, as well as the hemolytic and non-hemolytic enterotoxins, Hbl and Nhe. Alignment of the promoter regions activated by PlcR revealed a highly conserved palindromic sequence (TATGNANNNNANCATA) designated as the PlcR box. The −10 region of the PlcR-regulated promoters resembles that of promoters bound by an RNA polymerase associated with the Sigma A factor. However, the −35 region of these promoters significantly diverges from the −35 sequence generally recognized by Sigma A. The transcription start site of the PlcR-regulated genes can be located at various positions downstream from the palindromic sequence (from a few nucleotides up to about 200 nucleotides in the case of the *hbl* and *nhe* operons). Proteomic and transcriptomic analyses allowed determination of the complete PlcR regulon in *B. cereus* and *B. thuringiensis*. In these bacteria, PlcR positively controls the expression of 45 genes and accounts for about 80% of the secretome during early stationary phase [12,40]. Forty of the PlcR-controlled proteins are exported and the functions of these proteins are related to food supply and virulence (phospholipases, proteases, hemolysins, and toxins), cell protection and environment-sensing.

**Figure 2:**
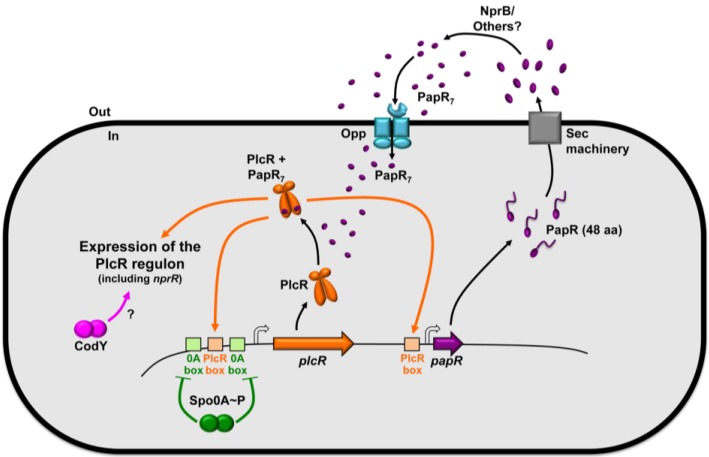
Schematic representation of the PlcR-PapR QS system, its regulation and its activity. The product of the *papR* gene, belonging to the PlcR regulon, is a 48 amino-acid peptide. PapR is secreted, probably via the Sec machinery, processed by the NprB protease and presumably other peptidases. PapR is found in the extracellular compartment and in the bacterial cytoplasm mainly as a seven amino-acid peptide. It is imported in the cell via the oligopeptide permease Opp. Once inside the cell PapR binds to PlcR allowing it to activate the expression of 45 genes composing the PlcR regulon. *plcR* is autoregulated and under the negative control of Spo0A-P. CodY positively controls the expression of the PlcR-dependent genes via a yet unknown mechanism.

The presence of a small PlcR-regulated gene, *papR*, located 70 bp downstream from *plcR* and the beginning of *plcR* expression being tied to the onset of stationary phase hinted to a possible regulation via a QS mechanism. Deletion of *papR* abolishes the activity of PlcR and results in a significant reduction of virulence [41]. The *papR* gene product is secreted, diffusible in the extracellular medium and processed mainly as the ADLPFEF heptapeptide, corresponding to the carboxy-terminal end of the peptide [32,42]. The peptidic sequence of PapR contains a putative signal for secretion by the general secretion Sec pathway, but export of the peptide via this system has not been demonstrated. Maturation of PapR depends on NprB, a protease that belongs to the PlcR regulon [43]. However, complementation experiments using the supernatant of a *plcR* mutant suggested that other proteases are involved in this process (our unpublished results). The processed form of PapR is subsequently imported into the bacterial cells via the oligopeptide permease Opp [44]. Once inside the bacteria, PapR interacts with PlcR, allowing its binding to the PlcR boxes [41]. Therefore, the PlcR-PapR complex forms a quorum sensing system in which PlcR is the quorum sensor and PapR the signaling molecule. This system allows the coordination of gene expression to cell density. Figure 2 summarizes the regulation of the expression of the *plcR* gene and the activity of its product in association with PapR. Recent structural data explained the activation mechanism of PlcR: peptide binding in the TPR domain of the protein results in more flexible HTH domains; this conformational change allows the formation of a drastic kink in the helix linking the two domains upon DNA binding. This mechanism allows the binding of the HTH domains of the PlcR dimer in the two half sites of the palindromic PlcR box [36].

The activation of PlcR by PapR is strain-specific, and this specificity is determined by few residues of the signaling peptide and of the quorum sensor [42,45]. Alignment of PapR sequences from various strains of the *B. cereus* group, and PlcR activation assays using various PapR have led to the identification of four specificity groups. The PlcR regulon of a strain belonging to a given specificity group can be activated by its cognate signaling peptide. However, this PlcR regulon is not, or poorly, activated by the strains producing PapR molecules from other specificity groups. This suggests a co-evolution of PlcR and PapR that may reflect niche specificities.

Recent reports have shown that the global stationary phase regulator CodY is required for expression of the PlcR regulon [46,47]. However it is not known at what stage of the activation process this pleiotropic regulator is required. CodY has previously been shown to control virulence in several other Gram-positive bacteria such as *B. anthracis*, *S. aureus*, and *Listeria monocytogenes* [48,49]. The YvfTU two-component system is also involved in *plcR* expression [50]. However, the inducing cues for signal transduction in this system are unknown and direct activation of *plcR* expression by the response regulator was not demonstrated. It was also shown that transcription of *plcR* is dependent on the growth medium and is repressed by the master regulator of sporulation, Spo0A [51]. It results that PlcR is not active in *B. thuringiensis* cells growing in a sporulation-specific medium, and that the PlcR-regulated genes are only expressed in bacteria growing in rich medium, like the LB medium, or in insect larvae [15].

## 3. Survive: NprR and Necrotrophism

In sporulation-specific medium, the neutral protease NprA is the major extracellular protein massively produced by bacteria of the *B. cereus* group [52,53]. Expression of *nprA* is controlled by NprR, a regulator whose transcriptional activity depends on the NprX signaling peptide SKPDIVG [53]. Contrary to PlcR, NprR is active in sporulation-specific growth conditions. A recent report has demonstrated that NprX binds to the TPR domain of NprR and induces a switch from a dimeric apo form to a tetrameric form active as a transcriptional regulator [37]. In association with NprX, NprR activates the transcription of at least 41 genes [15]. NprR-NprX binds to a DNA region encompassing the −35 box in the promoter region of *nprA* [53], but additional experiments are needed to precisely determine the DNA sequence recognized by the complex. The NprR regulon includes genes encoding degradative enzymes (proteases, lipases and chitinases) and a lipopeptide (kurstakin) involved in biofilm formation. Using insect larvae as an infection model, it was shown that NprR is not involved in the virulence of the bacteria. However, the NprR regulon is essential for the survival of the Bacilli in the cadaver of infected insects [15]. Therefore, the NprR-NprX system allows the bacteria to survive and eventually to sporulate in the host cadaver, thus, improving their ability to disseminate in the environment.

**Figure 3:**
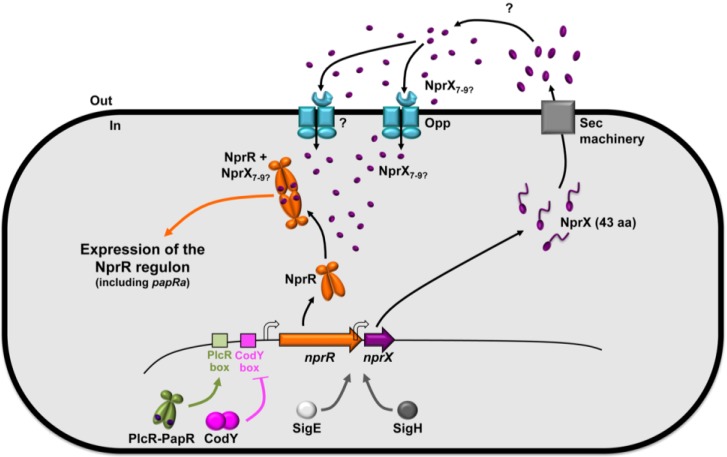
Schematic representation of the NprR-NprX QS system, its regulation and its activity. The product of the *nprX* gene is a 43 amino-acid peptide. NprX is secreted, probably through the Sec machinery and processed via an uncharacterized mechanism. The physiological form of NprX has not been determined. However, the minimal active form is seven amino-acids long. It is imported in the cell via the oligopeptide permease Opp and presumably another oligopeptide permease system. Once inside the cell, NprX binds to NprR allowing it to activate the expression of at least 41 genes composing the NprR regulon. Transcription of *nprR* is activated by PlcR-PapR and is under the negative control of CodY. *nprX* is co-transcribed with *nprR*, but also possesses a promoter under the control of SigH and SigE.

Figure 3 illustrates the regulation of the *nprR* and *nprX* genes as well as their activity. The *nprR* gene is located upstream from *nprA* and is followed by the *nprX* gene, which is co-transcribed with *nprR*. Contrary to *plcR*, the transcription of *nprR*-*nprX* is not autoregulated and is negatively controlled by CodY during the exponential growth phase. This control by CodY presumably links the expression of the NprR regulon to nutrient availability. When nutrients are available, CodY is active and represses expression of *nprR*. At the onset of the stationary phase, the expression of *nprR* is activated by PlcR, linking the virulence stage to the necrotrophic lifestyle [54]. However, two additional promoters, depending on Sigma H and Sigma E, direct transcription of *nprX*. This may allow a prolonged production of the NprX peptide to ensure expression of NprR-regulated genes during the sporulation process, while transcription of *nprR* decreases. Because it presents a typical export signal sequence, NprX is also likely to be exported via the general secretory Sec pathway. The maturation mechanism and the physiological size of the active form of the peptide have not been reported. However, peptides ranging from seven to nine amino-acids allow activation of NprR [37]. Interestingly, and in contrast to PapR_7_, the active form of NprX corresponds to a central part of the *C*-terminal region of the pro-peptide, suggesting a different maturation process. Characterization of the factors involved in the step allowing entry of the mature form in the cell is under investigation. Preliminary results showed that several oligopeptide permease systems are responsible for the import of NprX [55], contrary to PapR that only requires Opp [44]. Similarly to the PlcR-PapR system, the NprR-NprX system is strain specific [53]. Based on the NprR and NprX peptidic sequences, seven pherotypes were defined within the *B. cereus* group with a range in the stringency of the specificity across the NprR-NprX pherotypes.

Using the insect model of intrahemocoelic infection, analysis of the expression kinetics of *nprA in vivo* revealed that transcription of this gene significantly increases after the death of the insect to reach a maximum 48 h after inoculation [15]. Interestingly, the PlcR-dependent *mbpE* gene is expressed earlier in the same conditions and its transcription reaches a maximum 24 h after infection. This shows that *mbpE* and *nprA* that reflect the activity of the two QS systems PlcR-PapR and NprR-NprX, respectively, are sequentially activated during infection.

## 4. Resist: Rap and Sporulation

It has been recently shown that a *B. thuringiensis* *sigmaK* mutant was able to survive in an insect cadaver, albeit to a lower level than a wild-type strain [15]. This suggests that survival of *B. thuringiensis* cells in a host cadaver is partially independent on sporulation. However, an efficient sporulation is the warranty of a better survival outside the host. The process of sporulation has been more extensively studied in *B. subtilis* than in *B. thuringiensis* or *B. cereus* and is the result of a complex differentiation pathway. The initiation of sporulation is regulated by Spo0A, the key transcription factor that controls early stationary phase and sporulation gene expression [18]. The activity of Spo0A is governed by the multicomponent phosphorelay consisting of five histidine kinases (KinA to KinE), the Spo0F response regulator and the Spo0B phosphotransferase [56]. The phosphoryl groups are transferred from the kinases to Spo0F (Spo0F-P) then to Spo0B (Spo0B-P), which finally phosphorylates Spo0A to generate Spo0A-P [57]. The Rap phosphatases modulate the level of Spo0A-P by dephosphorylating Spo0F-P in order to prevent early sporulation [58]. The balance of the action of kinases and phosphatases allows a gradual increase in the concentration of Spo0A-P until it reaches a threshold level above which sporulation is triggered [59].

The phosphatase activity of the Rap protein is regulated by the Phr signaling peptides that directly inhibit their activity through a QS system. Rap proteins are structured in TPR domains which mediate interactions with Spo0F-P or with the Phr inhibitor peptide [35,60]. The Phr peptide restores the transfer of the phosphoryl groups by displacing Spo0F~P from a preformed complex with Rap [61]. Despite the presence of a canonical signal peptide sequence, the involvement of the typical signal peptidases in processing the pre-propeptide could not be demonstrated [62]. The pro-peptide PhrA, but not PhrE, was shown to be matured by proteases of the subtilisin family [63]. The oligopeptide permease system (Opp) then allows the import of the signaling peptide into the cytoplasm of the bacteria [64,65]. This circuit ensures the appropriate timing for the phosphatase activity of the Rap protein in order to allow the bacterial population to sporulate efficiently.

The Phr-encoding genes are generally located downstream of the *rap* genes and are co-transcribed from the *rap* gene promoter [65]. Several regulators are involved in controlling the transcription level of these genes. For example, transcription of *rap*A-*phr*A is positively regulated by the response regulator ComA [66] and negatively affected by the transcriptional regulator CodY [67]. In addition, most of the *phr* genes have a second promoter controlled by the alternative Sigma H factor [68].

Rap proteins have not been studied in *B. thuringiensis* thus far. The Spo0F, Spo0B, and Spo0A proteins are present in all of the *Bacillus* species and the key amino acids that govern the molecular basis for recognition specificity between these proteins are highly conserved [69,70,71]. Five histidine kinases involved in the sporulation of *B. anthracis* were identified by analogy with the histidine kinases found in *B. subtilis* [72]. Moreover, an *oppB* mutation significantly reduced the sporulation efficiency in *B. thuringiensis* in rich medium [44]. This suggests that the mechanisms controlling the entry in sporulation are at least partially conserved between *B. subtilis* and bacteria of the *B. cereus* group.

Among the six putative Rap-Phr systems encoded in the *B. anthracis* genome only two are involved in the regulation of sporulation initiation with a phosphatase activity against Spo0F~P [73]. The BXA0205-BXA0205Phr system, which is associated with the strongest phenotype, is located on the pXO1 virulence plasmid. The phosphatase activity of the BXA0205 Rap protein is inhibited by the carboxy-terminal pentapeptide GHTGG of the BXA0205Phr peptide. The second system, BA3790-3791, is located on the chromosome and the active form of the Phr inhibitor corresponds to a central part of the peptide sequence but has not been clearly defined.

BLAST analyses identified eight putative Rap proteins presenting between 42% and 59% identity with the amino acid sequence of the *B. anthracis* BXA0205 Rap protein (Table 1). Moreover, open reading frames (ORF) encoding putative Phr-like peptides of 44 to 96 amino acids are located at the 3' end of each identified *rap* gene. The Phr peptide corresponding to the RapK protein is the only one that has not been annotated in the database. Analysis of each Phr peptide with the SignalP 4.1 or Phobius programs [74,75] indicated a putative signal peptide cleavage site (Table 2) typical of secreted proteins, as previously described for the Phr peptides of *B. subtilis* and *B. anthracis* [28,73]. The *B. anthracis* BXA0205-BXA0205Phr system could correspond to the RapK-PhrK or the RapF-PhrF systems in the *B. thuringiensis* strain 407. Similarly to the *bxa0205-bxa0205phr* genes, the *rapK*-*phrK* and *rapF*-*phrF* genes are plasmid-borne. The BXA0205 Rap protein shares 59% identity with RapK and 52% identity with RapF. Interestingly, the carboxy-terminal pentapeptide GHTGG, defined as the active form of the BXA0205Phr, is highly conserved in PhrK and PhrF (Table 2). The second *B. anthracis* Rap-Phr system, BA3790-3791, could resemble to the RapF2-PhrF2 system in the *B. thuringiensis* strain 407. The *rap*F2-*phr*F2 genes are located to the chromosome like the *ba3790-3791* genes and the BA3790 Rap protein shares 53% identity with RapF2. As the active form of the BA3791 Phr has not been clearly characterized, no conclusion could be drawn about similarities with PhrF2.

**Table 1:** *rap* genes in the *B.* *thuringiensis* strain 407 genome. Rap proteins have been identified by BLAST analysis with the amino acid sequence of *B. anthracis* BXA0205 Rap protein as a query.

Locus tag ^a^	Annotation ^b^	% Identity with BXA0205	Localization
BTB_6p00030	*rap*K	59%	pBTB_6p plasmid
BTB_78p00160	*rap*F	52%	pBTB_78p plasmid
BTB_c35690	*rap*F2	51%	chromosome
BTB_c10360	*rap*F1	47%	chromosome
BTB_9p00050	*rap*I	46%	pBTB_9p plasmid
BTB_c10810	*rap*I1	43%	chromosome
BTB_c17720	*rap*-like ^c^	45%	chromosome
BTB_502p04160	*rap*C	42%	pBTB_502p plasmid

^a,b^ As annotated in the *B. thuringiensis* strain 407 genome sequence (accession number CP003889 to CP003898, Sheppard, 2013); ^c^ Not annotated, personal annotation.

**Table 2:** Amino acids sequences of the putative Phr signaling peptides of *B. thuringiensis* strain 407 and the BXA205Phr of *B. anthracis*. The BXA205Phr pentapeptide inhibitor is highlighted in orange. The putative PhrK and PhrF penta-peptide inhibitors are highlighted in purple. The putative signal peptides are underlined.

Annotation ^a^	Sequence	Length ^b^
BXA0205Phr	MKKVMFSLIGLTAVFTFMFNASNVTDTQKALSEDKVVQYAH 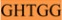	46
PhrK	MKKTILTLMGIITVFTLTLSNINTPKENKDPSIQKIMLMSD 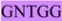	46
PhrF	MKKTLISLMGIVTILTFTIGLSNPGEVQKFIQTKVAASE 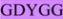	44
PhrF2	MIKKISSIVLGLSVLSIVSIGLNSSFTYQAGHADFPAPQRPDLVQSIDVSKDYNSTTTEKAL	62
PhrF1	MKRIISSSIGLIITVILLNGVNVSTDQLKVNTTSVIQYTHGEPWG	45
PhrI	MMKKFSLILIGVACTTGIFFSQFNNSIQTHDAKEKNDIIQQYAHGKDI	48
PhrI1	MKKFRLAIVGTALVGVLSIGFNSSFTNQAVNIGDTGGAPARPDYVNIGDTGGAPARPDYVNIGDTGGAPA	96
Phr-like ^c^	MKKISLAILTFTCILAFGFNNFTESQQAKQLPKWDTDKQETHADL	45
PhrC	MKKFKIALLGFVSVAVLSLGLNSGTETQKASSVTESPTHVIYYSHADVW	49

^a^ As annotated in the *B. anthracis* A2012 (accession number NC_003980, Biongiorni 2006) and in the *B. thuringiensis* 407 genome sequence (accession number CP003889 to CP003898, Sheppard 2013); ^b^ Length of the entire peptidic sequence; ^c^ Not annotated, personal annotation.

This comparative analysis with the Rap-Phr systems of *B. anthracis* suggests that at least three different Rap-Phr systems (RapK-PhrK, RapF-PhrF and RapF2-PhrF2) could be involved in the control of the initiation of sporulation in *B. thuringiensis* strain 407. Further studies will be needed to examine the involvement of these three systems in the sporulation process and to understand the role of the other Rap-Phr pairs in the lifecycle of *B. thuringiensis*.

## 5. PlcRa and Other PlcR-Like QS Systems in *B. thuringiensis*

Analyses of the *B. thuringiensis* genome revealed the presence of three PlcR-like proteins on the basis of their primary peptidic sequence homology to PlcR. PlcRa, PlcRb and PlcRc display about 29% identity and 50% similarity to PlcR and are 85% identical. The *plcR*-like genes are located in three different *loci* on the chromosome of *B. thuringiensis* strain 407. A small ORF, *papRa*, encoding a peptide with a putative signal sequence, is located upstream from the *plcRa* gene. In contrast, *plcRb* and *plcRc* are not associated with such ORF. A homology model for PlcRa, PlcRb and PlcRc was constructed, based on the X-ray structure of the PlcR dimer [76], and our unpublished results. Each PlcR-like monomer is composed of an HTH domain in its N-terminal region, followed by a linker helix that connects the HTH to the five TPRs and is at the interface between the two monomers. The TPR domain of the PlcR-like proteins is arranged as described for PlcR, and forms a hydrophobic pocket that can potentially accommodate a peptide.

As hypothesized, PlcRa is activated by the product of the *papRa* gene, a secreted signaling peptide [76]. As described for PapR, a typical Gram-positive N-terminal signal peptide was identified for PapRa using the SignalP program [74]. Based on a sequence alignment with the PapR peptide, the CSIPYEY fragment, PapRa_7_, was proposed as the PlcRa cognate signaling peptide. PapRa_7_ corresponds to an internal region of the carboxy-terminal part of PapRa, contrary to PapR_7_ whose mature form corresponds to the C-terminal end of PapR. Biochemical and genetic analyses demonstrated the role of PapRa_7_ in PlcRa activity. Purified PlcRa specifically binds to a PlcRa-controlled promoter and its binding requires the presence of synthetic PapRa_7_. Addition of PapRa_7_ to growing cells or overexpression of *papRa* activates the expression of *abrB2*, a PlcRa-dependent gene, at the onset of stationary phase [76]. Interestingly, *papRa* is included in the NprR regulon [15]. Preliminary results also suggest that PlcR is indirectly involved in the activation of PlcRa (our unpublished data). Data about the maturation and import of PapRa have not been reported yet. Analysis of 13 *B. thuringiensis* and 13 *B. cereus* complete genomes sequences revealed that the PapRa heptapeptide is conserved in all strains suggesting that there is only one pherotype associated to this QS system, in contrast to PapR and NprR.

Transcription of *plcRa* is not autoregulated and is triggered at the beginning of stationary phase in rich medium. Analysis of the transcriptome of a *plcRa* mutant compared to that of the wild-type strain showed that 68 and 49 genes were up- and down-regulated, respectively, at the onset of stationary phase [76]. PlcRa notably positively regulates the transcription of genes involved in regulation and synthesis of cysteine and in peroxide stress resistance. Most of the PlcRa-positively controlled genes belong to regulons that are involved either directly (PerR, OhR) or indirectly (CymR) in the responses to peroxide and disulfide stresses in *B. subtilis* [77,78] and in *B. cereus* [79]. The *plcRa* mutant is more sensitive to hydrogen peroxide (H_2_O_2_) and diamide stresses than the isogenic wild-type strain and cystine addition to a *∆plcRa* culture improved H_2_O_2_ stress resistance [76]. It has previously been shown in *B. subtilis* that modifications in the intracellular concentration of cysteine lead to increased sensitivity to oxidative stresses [77,78]. Cysteine is the direct precursor of low-molecular weight thiol molecules such as bacillithiol and Coenzyme A. In *Bacillus* species, these molecules are the key actors in the maintenance of cytosolic redox balance and in adaptation to the presence of reactive oxygen species [78,80,81]. This study demonstrated for the first time the existence of regulatory connections between cysteine metabolism and the oxidative stress responses in *B. cereus*.

*plcRa* inactivation had no significant effect on sporulation in many common laboratory conditions. The involvement of PlcRa in the cellular response to various stresses that might be encountered during infection suggests that the PlcRa regulon might have a role during the lifecycle of *B. thuringiensis* in its host. This is currently under investigation. The role of *plcRb* and *plcRc* in the physiology of *B. thuringiensis* is yet to be determined.

## 6. Conclusions

**Figure 4:**
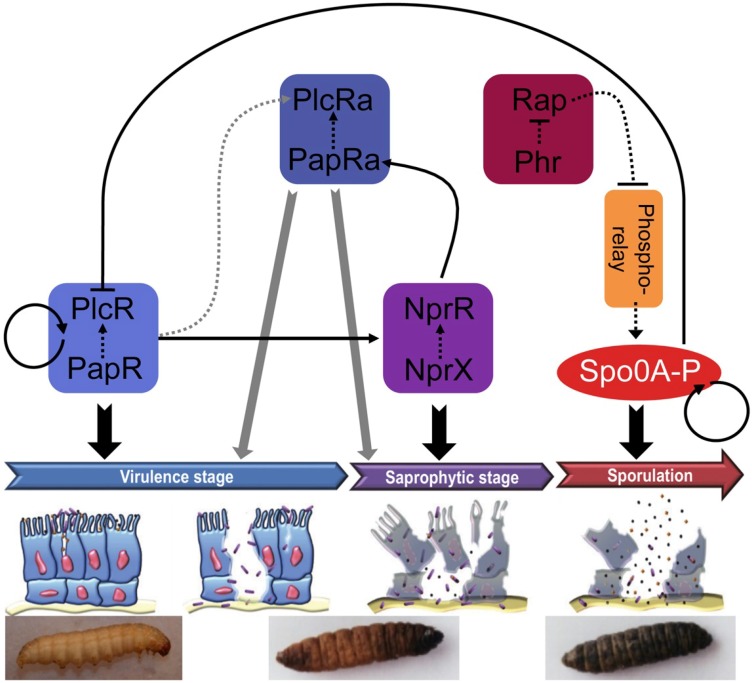
Sequential activation of the QS systems throughout the infectious process of *B. thuringiensis* in the insect. Solid lines represent a transcriptional effect. Dotted lines represent a peptide- or protein-protein interaction. The grey lines symbolize a putative effect. Arrows and blunt lines represent a positive or a negative effect, respectively. The bottom panels are pictures of *Galleria mellonella* 6th instar larvae, the model insect used for the infection experiments. From left to right: live larva, dead larva partially melanized, dead larva entirely melanized. Description of the figure is given in the main text.

The QS systems presented above are all intertwined via controls at the transcriptional or post-transcriptional level. We propose the following model to illustrate these interconnections (Figure 4). This figure describes the sequential activation of the four RNPP sensors described in the review and the connection of these direct QS systems with the physiological stages of the bacteria throughout the infectious process. During the early infection stage, PlcR is activated by its cognate peptide PapR and the PlcR-PapR complex switches on the virulence properties of the bacteria, resulting in the death of the insect [41]. In addition, PlcR is indirectly involved in the activation of PlcRa at the post-transcriptional level (our unpublished results). PlcRa controls cysteine metabolism and resistance to oxidative stress [76] that might be involved in resistance to stresses encountered during the infectious cycle. However, the role of this latter communication system in infection has not been demonstrated yet. PlcR-PapR also activates the expression of *nprR* and *nprX* [54]. The NprR-NprX complex activates genes involved in necrotrophism (saprophytic stage), thus allowing the bacteria to survive in the insect cadaver [15]. NprR-NprX also activates the transcription of *papRa*, a gene encoding a peptide showing similarity with PapR and mapping just upstream from the *plcRa* gene, whose product activates PlcRa. All these communication systems control the fate and the physiology of the bacteria, thus, establishing a strong coordination between regulation of gene expression, cell development and infection. In addition, we hypothesize that PapR and/or PapRa activate two other PlcR paralogs of unknown function (designated PlcRb and PlcRc) that are not associated with signaling peptides. Altogether these results show that the pathogenic and necrotrophic lifestyles of *B. thuringiensis* and *B. cereus*, and presumably their sporulation and resistance to oxidative stress, are tightly controlled by four QS systems acting sequentially during the infection process.

*B. cereus* and *B. thuringiensis* produce a lot of extracellular proteins and peptides [81]. For example, the *B. cereus* ATCC14579 supernatant contains more than 45 µg of proteins per mL, 2 h after entry into stationary phase in LB medium [40]. New signaling peptides that might be involved in various processes, yet to be described, are likely to be found in the extracellular environment of these bacteria.
